# Catalytic Promiscuity of the Radical *S*-adenosyl-*L*-methionine Enzyme NosL

**DOI:** 10.3389/fchem.2016.00027

**Published:** 2016-06-22

**Authors:** Wei Ding, Xinjian Ji, Yongzhen Li, Qi Zhang

**Affiliations:** Department of Chemistry, Fudan UniversityShanghai, China

**Keywords:** promiscuity, evolution, metalloenzyme, enzyme engineering, biosynthesis

## Abstract

Catalytic promiscuity plays a key role in enzyme evolution and the acquisition of novel biological functions. Because of the high reactivity of radical species, in our view enzymes involving radical-mediated mechanisms could intrinsically be more prone to catalytic promiscuity. This mini-review summarizes the recent advances in the study of NosL, a radical *S*-adenosyl-*L*-methionine (SAM)-dependent *L*-tryptophan (*L*-Trp) lyase. We demonstrate here the interesting chemistry and remarkable catalytic promiscuity of NosL, and attempt to highlight the high evolvability of radical SAM enzymes and the potential to engineer these enzymes for novel and improved activities.

It has long been assumed that enzymes have evolved to carry out specific functions with specific substrate recognition. While this assumption remains true in most cases, the past 2 decades have seen a dramatically growing number of examples where multiple functions are associated with single biomolecular entities (O'Brien and Herschlag, 1999; Copley, 2003, 2015; James and Tawfik, 2003; Bornscheuer and Kazlauskas, 2004; Khersonsky et al., 2006; Nobeli et al., 2009; Humble and Berglund, 2011; Pandya et al., 2014; Pabis and Kamerlin, 2016). The term “promiscuity” has been commonly used in biochemistry to describe enzymes that catalyze more than one reaction. The ability of enzymes to catalyze the same type of reactions on a series of substrates is referred to as substrate promiscuity or substrate ambiguity, whereas catalytic promiscuity means an enzyme catalyzes different chemical transformations via different reaction mechanisms (O'Brien and Herschlag, 1999; Copley, 2003; James and Tawfik, 2003; Bornscheuer and Kazlauskas, 2004; Khersonsky et al., 2006; Nobeli et al., 2009; Humble and Berglund, 2011; Pandya et al., 2014; Copley, 2015; Pabis and Kamerlin, 2016). Although the promiscuous activities usually are not of physiological relevance and were found by accident in biochemical analyses, they provide an important impetus for enzyme evolution, such as conferring selective advantages to members of a population where the promiscuous activities are beneficial for organismal fitness.

Radicals are commonly present in biochemistry and participate in myriads of cellular signaling and metabolic processes (Stubbe and van Der Donk, 1998; Frey et al., 2006; Buckel and Golding, 2012). Featuring the unpaired valence electrons, radicals are typically highly reactive, playing a pivotal role in many chemically challenging reactions by overcoming high kinetic and/or thermodynamic barriers. The high reactivity of radical species, on the other hand, can potentially lead to various side reactions, which, from a biochemistry point of view, are promiscuous reactions. Arguably, enzymes involving radical-mediated mechanisms are intrinsically more prone to promiscuity.

Among the most prominent radical enzyme families is the radical *S*-adenosyl-*L*-methionine (SAM) superfamily, which is a large and rapidly growing enzyme superfamily currently containing over 100,000 predicted members (Sofia et al., 2001; Frey et al., 2008; Booker and Grove, 2010; Vey and Drennan, 2011; Broderick et al., 2014; Wang et al., 2014). Radical SAM enzymes are found in all domains of life and believed to be among the earliest biological catalysts on earth (Frey et al., 2008; Broderick et al., 2014). These enzymes share a common mechanism for radical generation, utilizing a [4Fe–4S] cluster to bind SAM and reductively cleave its carbon-sulfur bond to produce a 5′-deoxyadenosyl (dAdo) radical. This primary carbon alkyl radical is highly reactive and initiates a remarkably diverse variety of reactions relevant to DNA repair, RNA and protein modification, and the biosynthesis of vitamins, coenzymes, and natural products (Bandarian, 2012; Zhang et al., 2012; Fluhe and Marahiel, 2013; Byer et al., 2015; Ding et al., 2015; Jarrett, 2015; Lanz and Booker, 2015; Mehta et al., 2015; Stojkovic and Fujimori, 2015; Yang and Li, 2015; Benjdia and Berteau, 2016; Hu and Ribbe, 2016; Landgraf et al., 2016). To achieve a specific catalytic outcome and avoid unwanted side reactions, the radical intermediates are presumably controlled by delicate van der Waals interactions in the enzyme active site, as exemplified by a prototypic radical SAM enzyme lysine 2,3-aminomutase (Lees et al., 2006; Horitani et al., 2015). Because of the intrinsic high reactivity of the radical intermediates, it may not be surprising that permutation of the van der Waals interactions in the enzyme active site by using unnatural substrates can result in catalytic promiscuity. An extensively-studied example is the radical SAM enzyme DesII involved in the biosynthesis of TDP-*D*-desosamine (Szu et al., 2009; Ruszczycky et al., 2012). DesII is a deaminase with regard to its native substrate TDP-4-amino-4,6-didoexy-*D*-glucose, but it acts as a dehydrogenase on an unnatural substrate TDP-*D*-quinovose, in which the C4 amino group is replaced with a hydroxyl group (Szu et al., 2009; Ruszczycky et al., 2012). In addition, DesII acts both as a dehydratase and a C3 epimerase on an unnatural substrate TDP-*D*-fucose (Ko et al., 2015), demonstrating its remarkable catalytic promiscuity.

Recent studies on the radical SAM enzyme NosL indicated that the catalytic promiscuity of radical SAM enzymes could be even more intriguing. NosL catalyzes the carbon-chain rearrangement of *L*-Trp (**1**) to produce 3-methyl-2-indolic acid (MIA, **4**) (Figure 1A), which is a key intermediate in the biosynthesis of a clinically interesting thiopeptide antibiotic nosiheptide (Yu et al., 2009; Zhang et al., 2011; Zhang and Kelly, 2012; Zhang and Liu, 2013; Just-Baringo et al., 2014). This enzyme shares sequence similarities with the *L*-tyrosine (*L*-Tyr) lyase family enzymes, including the hydrogenase-maturating enzyme HydG (Kuchenreuther et al., 2013; Duffus et al., 2014; Shepard et al., 2014; Dinis et al., 2015), thiamine biosynthesis protein ThiH (Martinez-Gomez et al., 2004; Kriek et al., 2007; Challand et al., 2010), and the F420 biosynthesis protein CofG (Decamps et al., 2012; Philmus et al., 2015); all of these enzymes are radical SAM-dependent and cleave the Cα-Cβ bond of *L*-Tyr. Similar to these *L*-Tyr lyases, *in vitro* assays showed that NosL cleaved the Cα-Cβ bond of *L*-Trp and produced significantly amount of 3-methylindole (**7**) (Zhang et al., 2011; Bhandari et al., 2015; Ji et al., 2015). These results lead to a hypothesis that MIA biosynthesis starts from scission of the *L*-Trp Cα-Cβ bond and then proceeds via an unusual recombination process that installs the carboxyl group on the C2 of the indole ring (Figure 1A; Zhang et al., 2011; Bhandari et al., 2015).

**Figure 1:**
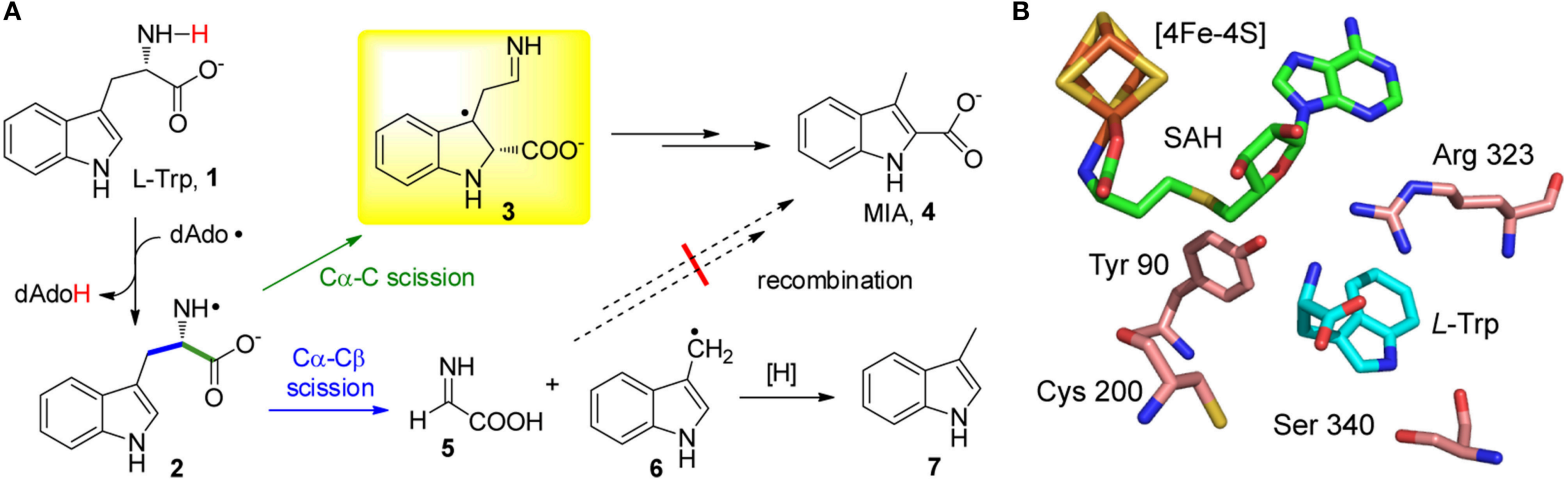
**NosL-catalyzed MIA biosynthesis. (A)** Proposed mechanism for NosL catalysis. The two fragmentation patterns of L-Trp are shown in green and blue, respectively. The key radical intermediate identified by Nicolet et al. (Sicoli et al., 2016) is shown in a yellow box. **(B)** The active site architecture of NosL (PDB ID: 4R33).

However, a recent study by Nicolet, Gambarelli, and coworkers demonstrated the presence of an unusual radical intermediate (**3**) in the NosL-catalyzed reaction, which contains a carboxyl group attached to the C2 of the indole ring (Figure 1A; Bridwell-Rabb and Drennan, 2016; Sicoli et al., 2016). Identification of this radical intermediate suggested that, in contrast to the previously proposed fragmentation-recombination mechanism, MIA biosynthesis likely proceeds via cleavage of the *L*-Trp Cα-C bond and subsequent migration of the carboxyl-fragment radical to the indole C2. This finding not only revealed the unusual radical chemistry in MIA biosynthesis but also highlighted the remarkable catalytic promiscuity of NosL, which diverts *L*-Trp into two parallel reaction pathways—one produces MIA (**4**) whereas the second produces glyoxylate (**5**) and 3-methylindole (**7**) (Figure 1A). Catalytic promiscuity is always associated with mutant enzymes and/or unnatural substrates, and the fact that a wild type enzyme exhibits two different types of activities with its genuine substrate is, to the best of our knowledge, unprecedented in enzymology.

The catalytic promiscuity of NosL was firstly revealed by an effort aiming to locate the hydrogen abstraction site in NosL catalysis (Ji et al., 2015), which was initially believed to be the indole nitrogen (Zhang et al., 2011) but later was suggested to be the amino group of *L*-Trp according to the NosL crystal structure (Figure 1B; Nicolet et al., 2014). An unnatural substrate 2-amino-3-(benzofuran-3-yl)propanoic acid (ABPA, **8**) was transformed to 3-methyl-2-benzofuranic acid (MBA, **9**) (Figure 2A), a benzofuran analog of MIA, thereby excluding the possibility that the dAdo radical-mediated hydrogen abstraction is from the indole nitrogen (Ji et al., 2015). Similar to this analysis, Begley et al. reached the same conclusion by using a thiophenyl substrate analog (**12**) and by using 1-methyl-*L*-Trp (**13**) in combination with mutagenesis (Figures 2B,C; Bhandari et al., 2015). Interestingly, when ABPA (**8**) was used in the reaction, the major product is neither MBA (**9**) nor 3-methylbenzofuran (**10**) but 2-(benzofuran-3-yl)ethanamine (BEA, **11**) a decarboxylated product of ABPA (Figure 2A), suggesting that NosL mainly serves as a non-oxidative decarboxylase on ABPA (Ji et al., 2015).

**Figure 2:**
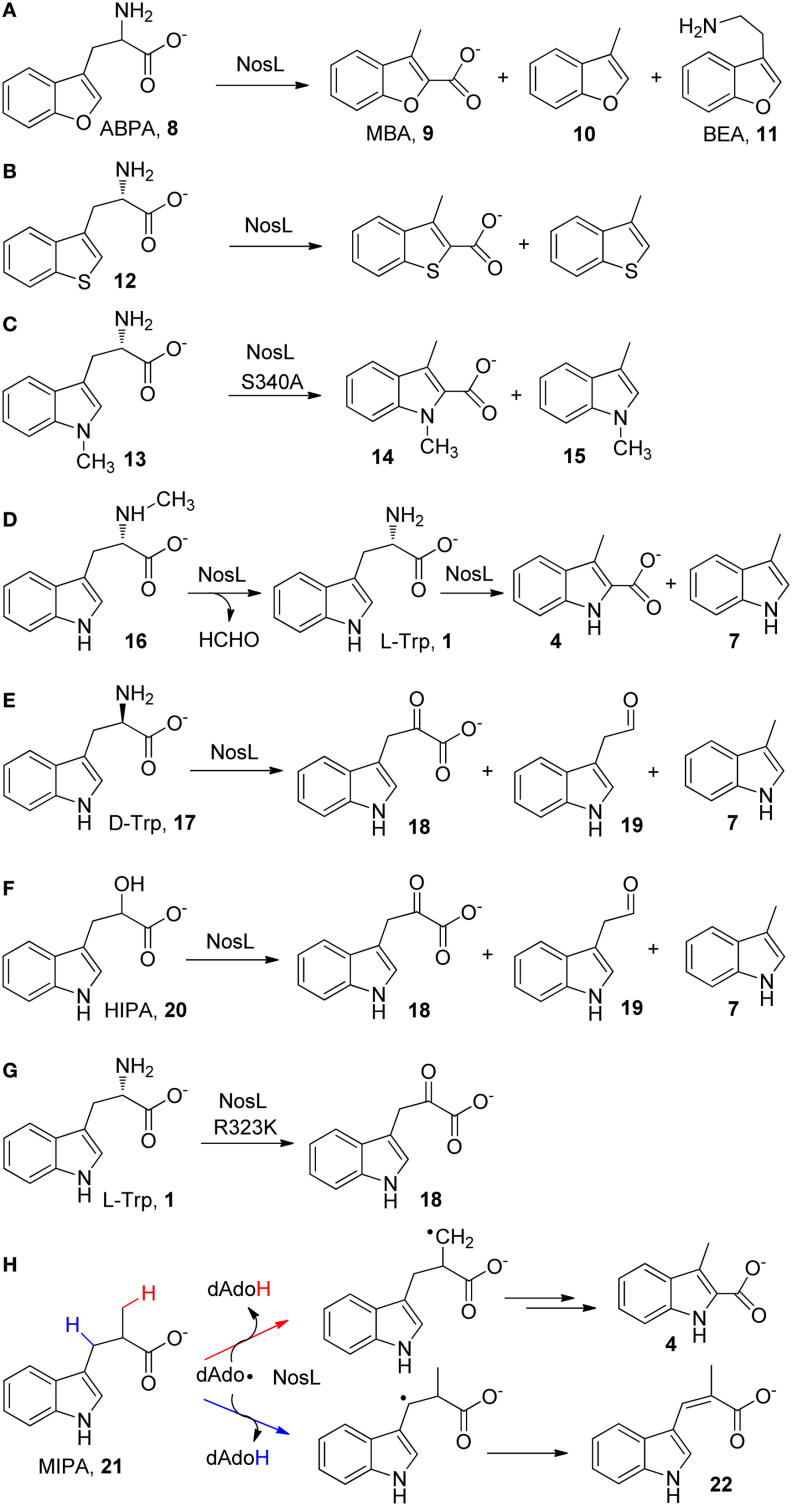
**Catalytic promiscuity of NosL. (A–H)** shows the *in vitro* reactions of various *L*-Trp analogs with NosL wild type or mutant enzymes. Simultaneous cleavage of both the Cα-Cβ and Cα-COO(H) bonds have been observed in most cases.

The catalytic promiscuity of NosL was further demonstrated by Begley and coworkers by using a series of substrate analogs (Bhandari et al., 2015). When N_α_-methyl-*L*-Trp (**16**) was used in the reaction, the enzyme acted as a demethylase and produced *L*-Trp (**1**), which was further transformed to MIA (**4**) and 3-methylindole (**7**) (Figure 2D). When *D*-Trp (**17**), the enantiomer of *L*-Trp, was used in the reaction, three different products corresponding to three possible β-scission reactions of the tryptophanyl radical (**2**) (i.e., deprotonation, decarboxylation, and Cα-Cβ bond cleavage) were observed in the reaction (Figure 2E). Similar β-scission reactions were also observed when 2-hydroxy-3-(indol-3-yl)propanoic acid (HIPA, **20**), an alcohol analog of Trp was used as a substrate (Figure 2F; Bhandari et al., 2015; Ji et al., 2015). Notably, while the decarboxylation reactions observed for several substrate analogs (Figures 2A,E,F) appear to be consistent with the mechanistic proposal by Nicolet et al. that involves Cα-C bond cleavage (Figure 1A), no decarboxylated product was observed in the reaction with *L*-Trp (Bhandari et al., 2015; Ji et al., 2015). The catalytic promiscuity of NosL was also demonstrated by a mutagenesis study, showing that change of an arginine residue (Arg 323) (Figure 2B) to lysine transformed NosL into an oxidative deaminase, which produced 3-(indol-3-yl)-2-oxopropanoic acid (**18**) from *L*-Trp and did not produce MIA and 3-methylindole anymore (Figure 2G; Bhandari et al., 2015).

More intriguingly, a recent study using 2-methyl-3-(indol-3-yl)propanoic acid (MIPA, **21**), a Trp analog in which a methyl group replaces the amino group, showed that the dAdo radical produced in NosL catalysis abstracted hydrogen atoms from both the methyl group and the Cβ of MIPA (**21**), thereby diverting MIPA into two different reactions that produce MIA (**4**) and a desaturated product (**22**), respectively (Figure 2H; Ji et al., 2016). These results demonstrated the conformational diversity of the enzyme active site.

In contrast to its remarkable catalytic promiscuity, NosL only exhibits moderate substrate ambiguity. This enzyme was shown to transform 5-fluoro-Trp and 6-fluoro-Trp to the corresponding fluorinated MIAs, and the latter compounds can be further incorporated into nosiheptide framework by downstream enzymes to produce fluorinated antibiotics (Zhang et al., 2011). In addition, as mentioned above, NosL converted the benzofuranyl and thiophenyl Trp analogs into the corresponding MIA analogs (Figures 2A,B; Bhandari et al., 2015; Ji et al., 2015). However, for many tested Trp analogs (5-hydroxy-*L*-Trp, 5-bromo-Trp, 6-methyl-Trp, to name a few), NosL failed to produce detectable amounts of the corresponding MIA analogs (Zhang et al., 2011). A NosL mutant carrying a Ser-to-Ala mutation (S340A) was shown to transform 1-methyl-*L*-Trp (**13**) to 1,3-dimethyl-2-indolic acid (**14**) and 1,3-dimethylindole (**15**) (Figure 2C; Bhandari et al., 2015).

An important question regarding NosL catalysis is how the same amino-centered tryptophanyl radical (**2**) is partitioned roughly equally into two parallel reaction pathways in the active site (Figure 1A). Like other members of the radical SAM superfamily, NosL adopts a triose phosphate isomerase (TIM) barrel fold (Nicolet et al., 2014), which is renowned for high evolvability of diverse enzyme functions (Zhang and DeLisi, 2001). The TIM barrel folds of the radical SAM superfamily enzymes have evolved to accommodate highly diverse substrates ranging from small molecules (e.g., *L*-Trp for NosL) to large biomolecules such as proteins and nucleic acids (Vey and Drennan, 2011). A key arginine residue (Arg 323) that directly interacts with *L*-Trp via a salt bridge resides in a loop region (Figure 1B; Nicolet et al., 2014), reflecting the plasticity of the enzyme active site that accommodates the substrate. When *in silico* scanning the conformation of the radical intermediate (**2**) (Figure 1A), Nicolet et al. found two thermally accessible stable conformations whose spin densities are consistent with scission of the Cα-Cβ and Cα-C bonds, respectively (Sicoli et al., 2016), suggesting the final catalytic outcome could be fine-tuned by subtle conformational change of the radical intermediates. Nicolet et al. also showed the key radical intermediate (**3**) mainly adopted two conformations that exhibited different spin relaxation properties (Sicoli et al., 2016), again highlighting the conformational diversity of enzyme active site and the flexible interactions between enzyme and radical intermediates, both of which account for the observed promiscuity of NosL.

It is generally believed that enzymes have evolved to satisfy cellular metabolism and their catalytic efficiencies stop improving unless the selection pressure remains. Apparently, NosL is not an efficient enzyme and far from being well evolved, a fact that is consistent with the role of NosL in biosynthesizing a secondary metabolite—although NosL is not an efficient MIA synthase, its activity is likely enough to support nosiheptide biosynthesis by the producer. From an evolutionary point of view, the ability of NosL to perform various promiscuous activities (i.e., a generalist) (Khersonsky et al., 2006; Khersonsky and Tawfik, 2010) could possibly render the organism selective advantages under certain circumstances, whereas being a highly efficient and specific enzyme (i.e., a specialisit) (Khersonsky et al., 2006; Khersonsky and Tawfik, 2010) is not necessary for the cell in this case. Given the intrinsically high reactivity of radical species and the fact that many radical SAM enzymes are involved in secondary metabolism, it is likely that the catalytic promiscuity of the radical SAM superfamily enzymes remains largely underestimated. The catalytic promiscuity of these enzymes could provide new opportunities to engineer novel biocatalysts with improved properties, which could be of enormous academic and industrial value.

## Author contributions

All authors listed, have made substantial, direct and intellectual contribution to the work, and approved it for publication.

## Funding

This work was supported by grants from the State Key Laboratory of Microbial Technology (M2015-1 to WD) and from the Chinese National Natural Science Foundation (31500028 to QZ). QZ would also like to thank the Thousand Talents Program for support.

### Conflict of interest statement

The authors declare that the research was conducted in the absence of any commercial or financial relationships that could be construed as a potential conflict of interest.
